# A Five-Year Retrospective Study on the Clinical Outcomes of Sacral Nerve Stimulation for Neuromodulation of the Lower Urinary Tract in a Tertiary Hospital

**DOI:** 10.7759/cureus.73626

**Published:** 2024-11-13

**Authors:** Anna Akpala, Tamara Lezama, Kehinde Jinadu, Mohammed Belal, Thomas King

**Affiliations:** 1 Urology, Queen Elizabeth Hospital Birmingham, Birmingham, GBR

**Keywords:** overactive bladder, retention of urine, sacral nerve stimulation, sacral neuromodulation, voiding diary (vd)

## Abstract

Aim: To assess clinical outcomes in terms of first to second-stage conversion rates, revision rates, and complications for all patients undergoing sacral neuromodulation of the lower urinary tract for the past five years at the Queen Elizabeth Hospital Birmingham.

Method: This is a retrospective observational study. Only patients with the first stage of sacral neuromodulation between January 2017 and January 2023 were included in the study. The data collected included age, type of first-stage trial, that is, percutaneous nerve evaluation (PNE) vs. tined lead, response after the first stage, whether a second trial was carried out, response after the second trial, indication for sacral nerve stimulation (SNS), complications, revisions, etc.

Results: 94% of the total (198) patients had PNE at the first trial, while 11 (6%) had tined lead. 129 (65%) patients had a positive response after the first trial, 49 (24%) had a negative response, and 20 (10%) had an equivocal response. 15 (8%) patients from the equivocal and negative response group had a second trial with a tined lead, with 53.3% (104) receiving a positive response, making our total conversion rate 69% (136). 100 (50%) patients had permanent implants fitted, 25 (13%) had complications, and 15 (7.5%) required revision. The indications for the revisions were lead migration (66, 33.33%), pain around the battery site (66, 33.33%), connection failure (40, 20%), battery charging problem (13, 6.67%), and device infection (13, 6.67%).

Conclusion: The conversion and complication rates are comparable to national standards and documentation in the literature, while the infection rates were lower. Higher conversion rates may be attained when tined lead is used at the first trial.

## Introduction

Sacral nerve stimulation (SNS) therapy is a reversible procedure that involves the stimulation of the sacral nerve, usually S3, by inserting the electrode lead into the corresponding foramen of the sacrum, which is then connected to a pulse generator [1]. Sacral neuromodulation can also be described as the stimulation of an isolated intact neuron, which brings about a physiological activity the organ is meant to perform; in this case, stimulation of the anterior roots of the sacral nerve brings about bladder contraction [2]. It is a well-established, safe, effective, minimally invasive advanced therapy that has widely been employed in the treatment of functional bladder abnormalities and bowel disorders refractory to conservative and medical therapy [3, 4]. Conservative management of functional bladder abnormalities is in the form of behavioral modifications such as pelvic floor exercises, reducing fluids in the evening, especially caffeinated and fizzy, smoking cessation, and bladder training, while medical therapy includes the use of alpha-blockers, bladder antispasmodics such as antimuscarinics and beta-3 adrenergic agonists such as mirabegron. When these fail, intradetrusor botulinum toxin A (BTX) injections or SNS are offered as third-line treatment options in these conditions [5,6]. 

SNS therapy was developed in 1982 by Tanagho and Schmidt and was approved by the Food and Drug Administration United States in the late 1990s for refractory voiding dysfunction urge incontinence, urgency-frequency syndrome, and idiopathic non-obstructive urinary incontinence [7]

Functional bladder abnormalities include neurogenic bladder, which can be secondary to multiple sclerosis, spinal cord injury, cauda equina syndrome, overactive bladder syndrome, and non-obstructive urinary retention. Overactive bladder syndrome (OAB), as defined by the International Continence Society, is defined as urinary urgency, usually with urinary frequency and nocturia, with or without urgency urinary incontinence [8,9]. Odeyemi et al. reported the overall prevalence of OAB-related symptoms in the United Kingdom to be 3.87 per 1000 persons, with an incidence of 2.79 per 1000 person-years [10]. Its prevalence has been reported to be as high as 16.5% in America [11].

The indications for SNS therapy include refractory overactive bladder with or without incontinence, neurogenic bladder from any cause including but not limited to multiple sclerosis, cerebral palsy, spinal cord injury, cauda equina syndrome, and non-obstructive urinary retention from any cause including Fowler’s syndrome.

In terms of efficacy, a meta-analysis by Tyler T et al. found that the success rates for the full implant and test phase stimulation were 84.2 and 66.2%, respectively [12]. This is similar to a report by Das et al. indicating that 76% of patients had a success rate over 6 months and at least 50% fewer episodes with SNS treatment, as opposed to 49% in the control group [13]. In another study, Siegel et al. revealed that 61% of patients who got SNS experienced a therapeutic success rate for overactive bladders, while 42% of patients who received standard medical therapy made the same claim [14]. A comparative study between SNS and BTX injections by Singh et al. reported that SNS resulted in fewer failure rates reported at six months compared to BTX for refractory OAB(31.8%. vs. 10.8%)[15].

The mechanism of action of sacral neuromodulation is not fully understood; however, the consensus is that it works by modulating the spinal cord reflexes and brain networks via peripheral and motor neurons rather than directly stimulating the detrusor muscle and sphincter [16,17]. One theory postulates that sacral neuromodulation is able to block afferent C fibres thereby preventing irregular voiding [18]. As published by Y wang et al., sacral neuromodulation in spinalized rats led to the reduction of bladder hyperreflexia by the inhibition of afferent C fibres, and this is supported by its use in neurogenic bladder conditions where C fibres are thought to be responsible for irregular voiding [3,18].

In OAB, SNS is believed to result in suppression of bladder reflex activity by activating afferent somatic prefrontal cortex and insula which it does by creating an electrical field around the sacral nerve roots [19].Its mechanism of action in urinary retention has been theorized to be via the inhibition of the guarding reflex, which in turn results in the reduction of the urethral sphincter tone, leading to voiding [17].

In this study, we examine the clinical outcomes of SNS therapy for the management of chronic lower urinary tract symptoms in a tertiary centre, the success rates of trials and conversion to permanent implants, and complications and revisions.

## Materials and methods

The method employed was a retrospective observational study. Patients included in the study were all patients with refractory overactive bladder, voiding dysfunction, or non-obstructive urinary retention who have failed to respond to conservative and medical therapy and have undergone primary SNS implantation with at least a one-year follow-up. Preoperatively, all patients were evaluated from history taking, physical examination, 72-hour bladder diaries, urodynamic studies, and international consultation on Incontinence Questionnaire-Urinary Incontinence Short Form questionnaire (ICIQ-UI) and occasionally, flexible cystoscopy.

Only patients who underwent primary first-stage treatment, either in the form of percutaneous nerve evaluation (PNE) or tined lead, between January 2017 and January 2023 in the Queen Elizabeth Hospital Birmingham with up to a one-year follow-up, were included in the study. Patients with incomplete or missing records were excluded. The patients underwent a staged procedure, with the first stage being the trial phase. This was either PNE or percutaneous tined lead.

Percutaneous tined lead

The first stage is performed in a prone position under local anesthetic and x-ray guidance in the operating theatre. The unilateral electrode in a sacral foramen is inserted, and then the response is checked following electrical stimulation. Since the procedure is performed under local anesthesia, patients can communicate the exact kind and level of stimulation they feel. This would normally be described as a feeling of pressure in the perineum, bellows, or movement in the toes and foot. Different voltage amplitudes are tested, and the lowest voltage is selected to produce the required stimulus as this would inform the pre-programming of the device. An adhesive dressing is applied to keep the lead in place and then connected to an external pulse generator device which is worn on a belt for the duration of the trial. The device is then activated by the urology clinical nurse specialist prior to discharge home.

The trial phase with the PNE lasts for two weeks, after which the lead electrode is removed. During the trial, the patients would note any improvement or change in their symptoms along with their frequency volume chart. The improvement in symptoms is based on patient-reported outcomes in terms of symptom improvement and a 72-hour frequency volume chart, which is compared to their pre-procedure parameters. Patient-reported outcomes of at least 50% improvement in symptoms evidenced on their 72-hour frequency volume chart is termed a positive response or successful trial. A failed or negative response is defined as less than 50% improvement in symptoms, while patients are said to have an equivocal response if there was a perceived improvement in symptoms but no change on the frequency volume chart. Patients who had a failed response or an equivocal response were offered a second trial with the percutaneous tined lead. Patients with a positive response proceeded to the second stage for permanent implant after the removal of the temporary lead.

Tined lead

A test phase using tined lead is similar to the PNE. Key differences include tined lead insertion being performed under general anesthetic without muscle relaxation and the tined lead device being able to be used for a much longer period of up to 6 weeks. Like the PNE, a unilateral electrode in the sacral foramen is inserted, and the response is checked by observing for bellows or plantar flexion of the toes, as these patients are under general anesthesia. The permanent lead is placed under X-ray guidance and then connected to the percutaneous extension, which is tunneled to the flank or buttock on one side, and the electrode is connected to an external stimulator. The device is then activated on the ward prior to discharge home. If the response has been equivocal after 2 weeks, the patient could have a longer trial of up to six weeks in total, at which point, if it is still equivocal or there is no response, this would be termed a failed trial phase and would not proceed to the permanent implant insertion.

Following a successful trial (a successful trial is defined as >50% improvement in symptoms as evidenced by their 72-hour voiding diary), a second-stage procedure is carried out. The site of the percutaneous extension is opened and disconnected from the lead. The lead is then attached to an implantable pulse generator (IPG) that is implanted in a battery pocket in the subcutaneous layer of the skin on the buttock or flank, which is powered by a primary or rechargeable battery. Note that the position of the lead has not been altered. The device is activated prior to discharge by the urology CNS, and the patients are followed up afterward, with an initial follow-up in six weeks, then six monthly, and then yearly.

**Figure 1:**
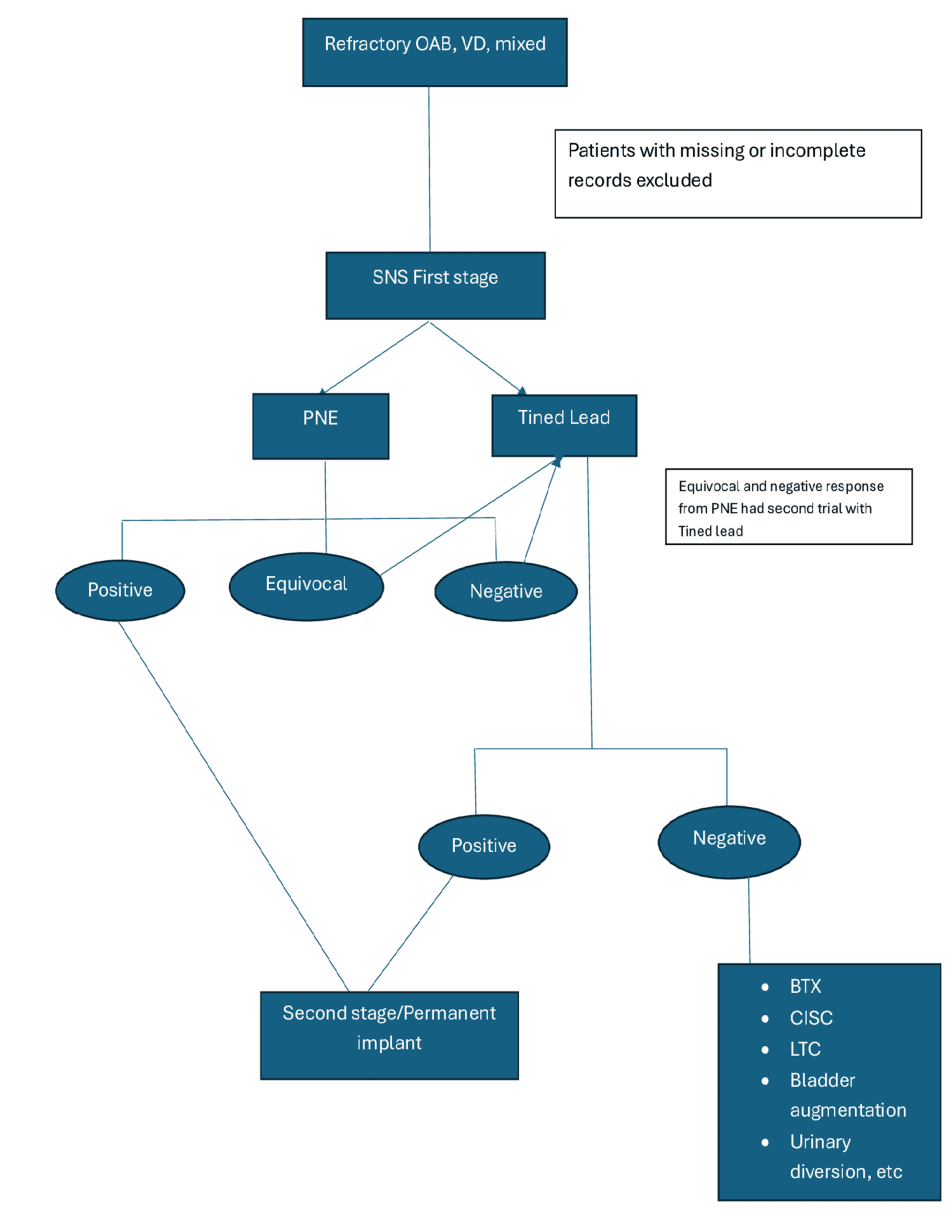
Flow chart of indications and response to SNS SNS: sacral nerve stimulation; PNE: percutaneous nerve evaluation; OAB: overactive bladder syndrome; VD: voiding dysfunction; BTX: intradetrusor botulinum toxin A injection; LTC: long-term catheter; CISC: clean intermittent self-catheterisation.

The indications for SNS in this study were divided into three broad categories: overactive bladder syndrome (which is further subdivided into idiopathic and neurogenic), voiding dysfunction (which is further subdivided into idiopathic and neurogenic), and mixed symptoms (voiding dysfunction and overactive bladder syndrome) as shown in Figure 1 below.

The patient details were obtained from the informatics department, and then parameters were sought from their electronic records, which were then entered into an Excel sheet. Following the quality assurance data review, data analysis was performed using IBM SPSS version 29, and statistical analysis was performed using Fisher's Exact test. The value at which the p-value was considered to be of statistical significance was set as <0.05.

## Results

The COVID-19 pandemic impacted the number of operations performed; however, a total of 198 patients were identified who had first-stage SNS within this time frame that fit into the criteria. 187 of them had PNE as the first trial, while 11 had tined lead. The value at which the p-value is considered to be significant in this paper is <0.05.

Age and gender

**Figure 2:**
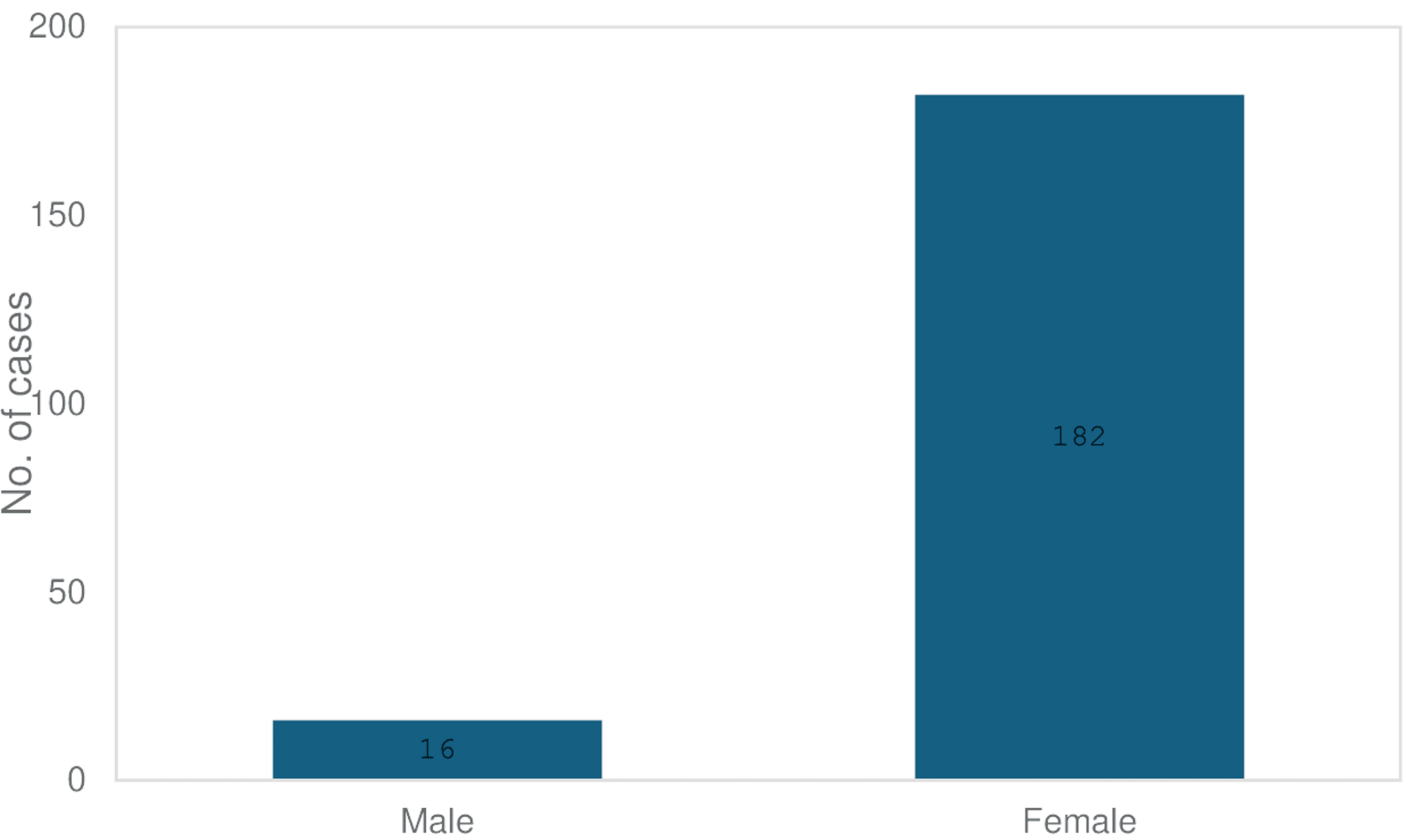
Gender distribution graph The graph represents the gender distribution of patients in the first-stage sacral nerve stimulation (SNS) cohort expressed in the number of patients (N).

There were 16 males and 182 females. The age range was between 19-82, with a mean age of 43.2 and a median age of 42. Four (2%) patients were aged <20, 64% (127) between 21-50, 34% (67) between 51-80, and only 1 patient (0.5%) was above 80 years. There were 182 females and 16 males in the trial stage. Figure 2 shows the sex distribution of the patients.

Type of first stage

**Figure 3:**
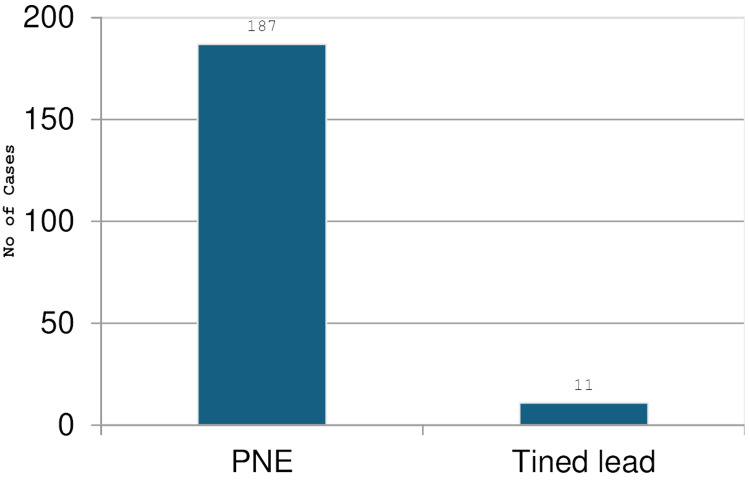
Graph showing distribution of patients in the trial phase The graph represents the distribution of patients in the trial phase expressed in number (N)

The first stage or trial was either performed using PNE or tined lead. Out of the 198 patients, 187 had PNE, and 11 had tined lead, as shown in Figure 3 below. It shows the distribution of patients in the trial phase.

Response to the first stage

A positive response is defined as an improvement in symptoms of >50%, as evidenced by the patient’s frequency volume chart. Overall, 65% (129) of the patients had a positive response following the first stage, 25% (48) had a negative response, and 10% (20) were equivocal, as seen in Table 1 below.

**Table 1:** Response distribution table among both groups following first stage. Comparison of response outcomes (positive, negative, equivocal) between PNE and tined lead groups in the first-stage trial (p-value=0.730, Fisher’s Exact Test, significant at p<0.05). The data has been represented as N and %.

First stage	Positive response (n, %)	Negative response (n, %)	Equivocal (n, %)	p-value
PNE	120 (63.8%)	48 (25.5%)	20 (10.6%)	0.730
Tined lead	9 (81.8%)	2 (18.2%)	0 (0%)	

Using Fisher’s Exact test, only positive and negative responses were analyzed, excluding equivocal responses due to the tined lead having 0 equivocal response (p=0.730), which indicates that this is not statistically significant.

Second trial

Following the first stage, some patients with negative and equivocal responses went on to have a second trial with the Tined lead. A total of 15 patients had a second trial, comprising 6 patients (40%) from the negative response group and 9 (60%) patients from the equivocal group. At the end of the second trial, 8 (53.3%) patients had a positive response.

Permanent implant

From this study, 69.19% (137) patients had a successful trial, and 72.99% (100) patients had permanent implants inserted. At the time of data collection, 25 patients were still on the waiting list for the procedure, while 12 declined Table 2.

**Table 2:** Conversion rate based on type of first stage. Conversion to the permanent implant for percutaneous nerve evaluation (PNE) and tined lead procedures in the first stage (p-value=0.202, Fisher’s Exact Test, significant at p<0.05). The data has been represented as N and %.

Type of first stage	Permanent implant (n=100)	Percent conversion (%)	p-value
PNE	91	48.66%	0.202
Tined lead	9	81.8%	

48.66% (91) of PNE trials resulted in a successful permanent implant, while 81.8% (9) of tined lead trials resulted in a successful permanent implant. Although the disparity in the numbers is almost 50%, p=0.202 indicating this difference is not statistically significant.

Indications of SNS

The indications of SNS in this paper were categorized into OAB, voiding dysfunction, or mixed. OAB made up 55.05% (109), while voiding dysfunction made up 40.40% (80), and only 4.5%(9) had mixed symptoms.

Complications

Table 3 below outlines the complications following permanent implants based on the type of first stage.

**Table 3:** Complications following permanent implant Complications by procedure type (PNE vs.Tined Lead) in patients with permanent implants. (p-value=0.632, Fisher’s Exact test, significant at p<0.05). The data has been represented as N and %.

Complication type	Total (n = 25)	PNE (n)	Tined lead (n)	p-value
Lead migration	7	7	0	0.632
Pain around battery site	6	6	0	
Connection failure	10	8	2	
Device infection	1	1	0	
Battery charging problem	1	1	0	
Complication rate		25.27%	18.18%	

The commonest complication following SNS in both groups were connection failure accounting for 40% (10). 28% (7) resulted from lead migration, 24% (6) from pain around the battery implant site, 4% (1) from battery charging problem and 4% (1)from device infection. Overall, 25.27% (23) of patients in the PNE group experienced complications, while 18.18% (2) in the tined lead group experienced complications. However, p= 0.632 indicates that the difference in complication rates between PNE and Tined Lead is not statistically significant.

Complications based on indications

The complications were also categorized based on indication, with 13 (52%) arising from the OAB group and 11 (44%) from the voiding dysfunction group, while 4% (1) accounted for the mixed group, as shown in Table 4 below. Using Fisher’s Exact test, p=0.931, there was no statistically significant difference in complication rates across the three groups.

**Table 4:** Complications based on indications of SNS Distribution of complications based on indication type (OAB, voiding dysfunction, and mixed) (p-value=0.931, Fisher’s Exact test. significant at p<0.05). The data has been represented as N. SNS-sacral nerve stimulation.

Indication	Complications (n)	p-value
OAB (idiopathic and neurogenic)	13	0.931
Voiding dysfunction (idiopathic and neurogenic)	11	
Mixed	1	

Revisions

Out of the 25 patients who had complications, only 15 required revisions. The indications for the revision are detailed in the graph below. One patient had a revision for a scheduled battery change as it had run out.

**Figure 4:**
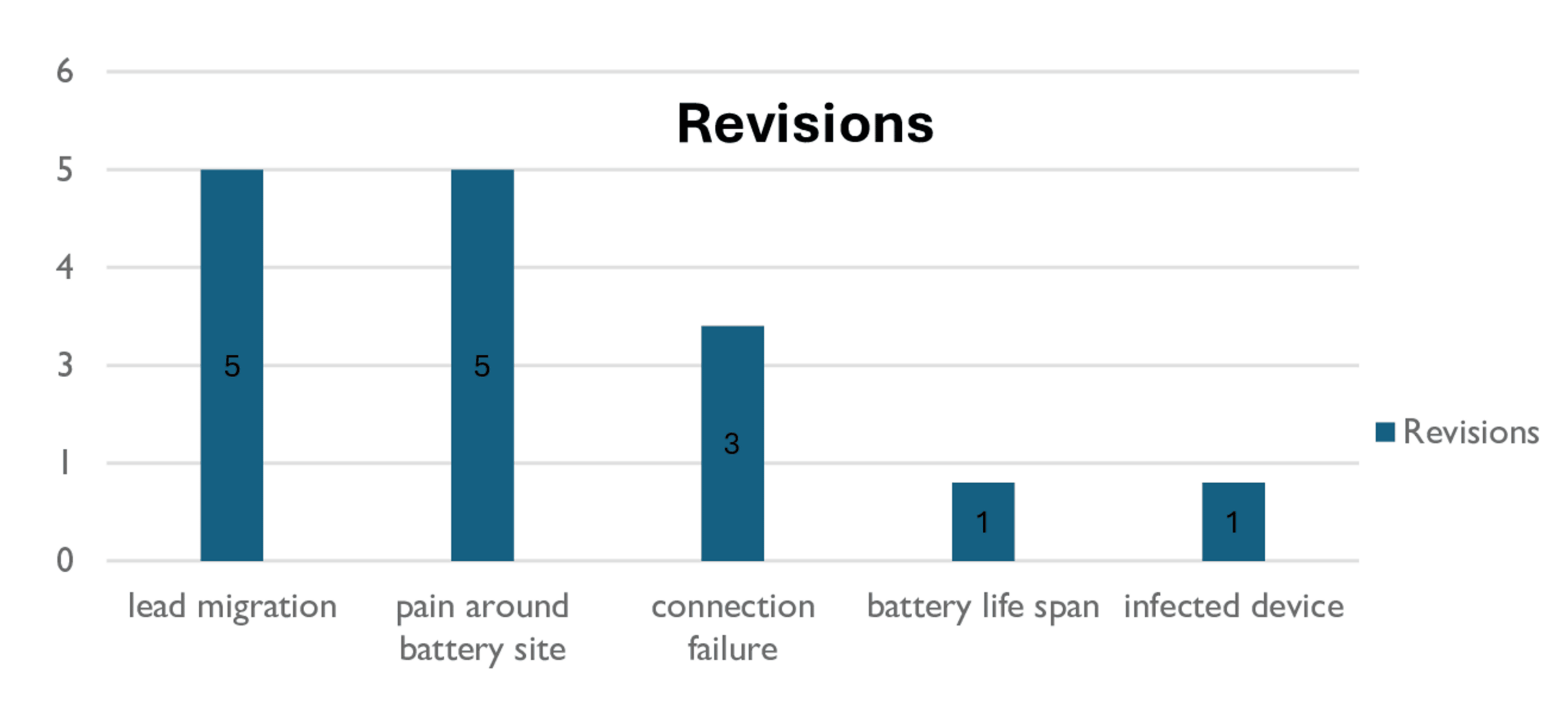
Indications for revision Indications for revision by complication type. (p-value=1.000, Fisher’s Exact test, significant at p<0.05). The data has been represented as N.

Revision rates

The indications for revisions were analyzed based on the type of first trial the patients had. 19.32% (13) of PNE patients underwent revision, while 18.18% (2) of tined lead patients underwent revision. Using Fisher’s Exact test, p=1 indicates no significant difference between the revision rates of PNE and tined lead, while an odds ratio of 0.928 indicates a slightly lower likelihood of revision with PNE but not statistically significant.

## Discussion

SNS efficacy

SNS as a management option for lower urinary tract conditions has been shown to be an effective therapeutic alternative to intermittent self-catheterization or indwelling suprapubic or transurethral catheters, thereby having a positive impact on patients' quality of life. Elterman D reported a success rate of 51-77% during the trial (PNE or tined lead) and 40-72% following permanent implantation for urologic chronic pelvic pain syndrome [20]. A systematic review by Ho et al. reported a success rate of SNS in the range of 42.5% - 100% (median=79.2%)[21]. Hernandez et al. demonstrated an overall efficacy of 60.4% and further subdivided their result based on indication for SNS. They showed a 77.8% improvement in symptoms for patients with OAB and only 40% improvement in patients with urinary retention [22]. Our study showed an efficacy of 69.19%, as out of the total 198 patients, 137 of them experienced a >50% improvement in their symptoms with SNS, which is comparable to those published by both Elterman D and Hernandez et al. The screening methods (tined lead vs PNE) for determining patient suitability for permanent SNS require further scrutinization. These disadvantages can negatively impact patient experience and have their own complications attached. With significant reductions in failure, adverse event, and surgical revision rates brought about by technological advancements in devices and modifications to surgical and testing methods, SNS has become a successful, minimally invasive treatment [23,24].

SNS indication

For patients with persistent bowel or voiding dysfunction who fail to respond to first- or second-line treatments (pharmacotherapy, diet and lifestyle changes, and pelvic floor muscle training), sacral nerve stimulation is now a well-established therapeutic approach. The main indications used in this study are overactive bladder and voiding dysfunction.

Overactive bladder

It was noted in the study that some participants in both the OAB and voiding dysfunction groups had neurologic conditions such as multiple sclerosis, cerebral palsy, and spinal cord injury. Even though our study did not assess the individual effects of SNS on OAB and voiding dysfunction, some studies have demonstrated that patients with urinary retention had a persistent favorable effect compared to patients with OAB, as reported by Hernández et al. [22].

Voiding dysfunction

The guarding reflex, a spinal-mediated reflex in which contraction of the external urethral sphincter lowers intravesical pressure and prevents urine leakage, was hypothesized to be inhibited in patients with nonobstructive urinary retention by SNS; consequently, bladder emptying may occur by lowering the tone of the sphincter [25]. In comparison to Hernandez et al. study [22], which showed that 40% of patients in the urinary retention group were able to micturate on their own without the need for a catheter, the mean number of catheterizations decreased.

According to Aboseif et al., SNS remarkably lowers health care costs associated with voiding in addition to improving quality of life. SNS implantation led to a 92% first-year reduction in costs associated with outpatient doctor visits, diagnostic and therapeutic procedures, and medication expenditure in a mixed group of patients who were not stratified based on the type of lower urinary tract dysfunction [24].

Tined lead vs PNE

Our study was heavily skewed, where 94% of patients had PNE at the first stage, and only 6% of the total 198 patients had tined lead. One study evaluating a tined lead for first-stage SNS showed that 74% of the patients in the tined lead group had a positive outcome [26], and this is comparable to the findings from our study, where 81.8% of the tined lead group had a positive response and only 64.2% in the PNE group achieved a positive response. This raises the possibility that tined lead is superior to PNE, although the difference is not statistically significant in our study (p=0.730). Some other studies have reported superior outcomes and conversion to permanent implant with tined lead in comparison to PNE [27, 16].

Tined lead also has the benefit of having a longer test phase (up to 6 weeks). The main disadvantage is that two operations require general anesthesia, unlike PNE, where local anesthetic and fluoroscopic guidance are sufficient. Lead migration is the main disadvantage and likely cause of reduced efficacy of PNE [28]. In our study, 28% of patients who had complications were due to lead migration and were in the PNE group. The issue of lead migration has been mitigated in recent years with the development of newer, more robust, and distensible models, which reduce the likelihood of lead migration.

Leong et al. showed a variety of ‘predictors’ that influenced the outcome of the first stage with tined lead, such as age, sex, mental health history, and urinary condition [27]. As the inclusion criteria in our study involved a range of lower urinary tract conditions, we should be cautious about applying our results and, thus, generalizations to all conditions. For future studies, it would be worth investigating the efficacy of tined lead versus PNE in relation to individual indications, gender, and age and comparing the complications and revision rates for each.

Complications of SNS procedure

White et al. reported a complication rate of 30.3% after a mean follow-up of 36.9 months following SNS with trauma and lead migration accounting for the majority of complications with a staggering 26.8% and 17.9%, respectively [29]. A review by the medical advisory secretariat published a complication rate of 33-50% with no record of permanent injury or death from the device [30]. Out of the total 198 patients in our study, 25 patients encountered complications, giving an overall complication rate of 12.6%, which is significantly lower than those published papers cited above.

According to a meta-analysis by Yang et al., complications from SNS include lead migration (1-21%), impaired defecation (4-7%), infection (4-10%), and regional pain at the implant site being the most commonest with an estimated incidence of 3-42% [31]. The complications encountered by patients in our study were subdivided into mechanical problems (lead migration, pain at the neurostimulator site), technical problems (connection failure), and infection. 

Mechanical

Lead migration: In a paper published by Coolen et al., lead repositioning accounted for 13% (10 patients) of the complications [28]. In our study, 7 (28%) of the complications were due to lead migration, which is also comparable to another study that demonstrated lead migration as the commonest complication, accounting for 13.8% [32]. This large difference between our study and the others may be due to PNE being used in a larger proportion of our study, and it has been shown that lead migration occurs less frequently with tined lead than with PNE [22]. Reprogramming, further lead fixing, or the contralateral insertion of a new lead are frequently used to address lead migration-related issues.

Pain at the battery site: Siegel et al. reported at 5 years of follow-up, 15% of complications were due to pain [14], while another paper published by Spilotros et al. reported that pain or discomfort at the implant site was the most common complication accounting for 15-42% of the complications [23] which is comparable to the 24% deduced from our study. The issue with pain and its impact on explantation or revision is the cause of the pain. Pain may be implant-related (implant position causing discomfort in some patients when sitting) or program-related (discomfort due to stimulation). Infection is also a cause of pain; however, this is an individual complication and will be discussed separately.

A simple way to differentiate between implant-related vs. program-related pain is by switching the device off. If due to the latter, reprogramming may rectify the issue, but if this is not possible, then revision will be required [22]. We find it is important to avoid creating too large a pocket when placing the IPG, and we endeavor to ensure this is as tight as possible as we feel this limits flipping and pocket pain.

Interestingly, according to our results, pain was not a factor in tined lead implantation. Again, due to the small number of patients implanted with a tined lead, this cannot be used for statistical purposes, but for further studies, it may be worth differentiating pain based on interval post-op as this would give a greater understanding of the cause of pain and the impact this has on the revision rates.

Technical

Connection failure/technical problems: In our study, 40% of complications were due to connection failure, which is higher than in other studies. The decline in these complication rates over time can be attributed mostly to technical and procedural improvements. Most technical problems could be solved by reprogramming the stimulator.

Infection/Infected Device

Another significant complication encountered is infection/infected device. According to Spilotros et al., pocket infection rates have been reported to be anywhere between 2-12%, and although this is infrequent, it often requires explantation of the device [23]. From our study, only one patient had a device site infection requiring revision, which is comparable to other published data. Although currently, there is no standardized antimicrobial prophylactic for sacral neuromodulation, due diligence should be taken, especially with regard to treatment duration and other possible related risks [25]. In our practice, preoperative intravenous gentamicin 240mg and 400-600mg teicoplanin and the use of Ioban dressing as infection prevention measures with standard aseptic techniques are implemented during the procedure. The French Association of Urology and the Neuro-Urology Committee suggested alternative prophylactic regimens, including intravenous cefotetan/cefoxitin 2 gm, amoxicillin-clavulanic acid 2 gm, or, in the case of allergy, vancomycin 15 mg/kg or clindamycin 600 mg (grade B recommendation) [23].

Revisions

Surgical revisions were done to relocate the stimulator for various reasons. There have been reports in the literature stating that the revision rates are between 9 and 33%, with pain being responsible for 15-42%, lead migration 4-21%, and infections 5.7-6.1% [33]. Our study has demonstrated a revision rate of 15%, with the main causes being due to lead migration (33.33%) and pain around the battery site (33.33%). Our overall revision rate of 15% is comparable to that reported in the literature with similar indications. In our study, only one patient required revision due to device infection, indicating we have very low device infection rates.

Limitations

The study’s limitations include its retrospective nature and the relatively small patient number, which predisposes to selection bias. Another limitation encountered is the lack of long-term results due to the lack of long-term patient follow-up after permanent implantation, so failures were not analyzed in this study. Due to the limited sample size for tined lead patients, it was difficult to give a true prediction of the efficacy of tined lead as a preferred option for the first stage. We were also unable to compare the efficacy of unilateral versus bilateral stimulation, as the patient involved in the study underwent unilateral stimulation, which is the most widely accepted method for SNS therapy. However, Schultz-Lampel et al. and some other studies suggest that bilateral stimulation obtained better results [34-36].

More extensive research with randomized multicentre studies with large data sets is needed to achieve a higher success rate and to comfortably conclude that tined lead is superior to PNE in terms of outcome. A randomized prospective study with a large patient number is required to compare outcomes following PNE and tined lead along with specific indications, gender, age, and severity of symptoms as this would aid in proper patient selection, but this would be challenging.

## Conclusions

Sacral nerve stimulation therapy has been shown to be a safe, effective, and minimally invasive treatment modality for refractory functional bladder abnormalities, with a success rate of up to 69% in our center, which is comparable to documentation in the literature. With more advancements in technology, complications of lead migration are being mitigated, and battery life now lasts up to 15 years, thereby decreasing trips to the operating department for battery change. Its cost and steep learning curve for the surgeon remain the limiting factor for use, particularly in low- and medium-income countries.
